# Diverse Presentations of Carcinoma Erysipelatoides from a Teaching Hospital in Australia

**DOI:** 10.1155/2012/134938

**Published:** 2012-12-23

**Authors:** Hui Ting Chow, Kim Tran, Ewan K. A. Millar, Jodi Lynch, Dedee F. Murrell

**Affiliations:** ^1^Department of Dermatology, St George Hospital, Sydney, NSW 2217, Australia; ^2^Department of Anatomical Pathology, South Eastern Area Laboratory Service, St George Hospital, Sydney, NSW 2217, Australia; ^3^University of New South Wales, Sydney, NSW 2052, Australia; ^4^Cancer Research Program, Garvan Institute of Medical Research, Darlinghurst, NSW 2010, Australia; ^5^Cancer Care, St George Hospital, Sydney, NSW 2217, Australia

## Abstract

Inflammatory breast carcinoma is a rare form of advanced breast cancer which carries a poor prognosis, even with treatment. Diagnosis is reached on clinical and pathological grounds; however, due to its propensity to mimic other conditions, it may often be delayed or missed by attending physicians. This case series describes four patients seen at our institution with a diagnosis of inflammatory breast cancer; 3 patients had a history of previously treated breast malignancy. In these cases, the emergence of a new breast lesion evaded initial diagnosis due to incomplete initial physical examination, falsely reassuring imaging results, lack of recognition that a cellulitis picture can resemble metastatic carcinoma, and inconclusive initial biopsy sections. These obstacles to achieve diagnosis serve to further worsen the prognosis by delaying the initiation of multimodality treatment which can improve survival. The purpose of our paper is to increase awareness among breast cancer specialists of the importance of undressing the patient for basic clinical examination of the breasts, recognition of the appearances of this type of local recurrence of breast cancer, and not to rely purely on ultrasound and mammography due to delay in diagnosis in some of our local cases. Sometimes deeper sections and repeat biopsies are needed to make the diagnosis.

## 1. Introduction

Inflammatory breast carcinoma, or carcinoma erysipelatoides (CE), is a rare and aggressive form of breast carcinoma with a rapidly progressive course. It has an incidence of 1%–6% of all breast cancer presentations in the United States, a rate which has doubled in the past 20 years [1]. Higher incidences are found in African Americans, who are also diagnosed at a younger age and have a poorer outcome [2]. Overall, CE constitutes 2% of all invasive breast tumours. The average age of onset is 45–54 years [3]. We report a series of four breast cancer patients who presented with inflammation of the skin around the chest, arm, or back, leading to an eventual diagnosis of inflammatory breast carcinoma.

## 2. Cases

### 2.1. Case 1

**Figure 1:**
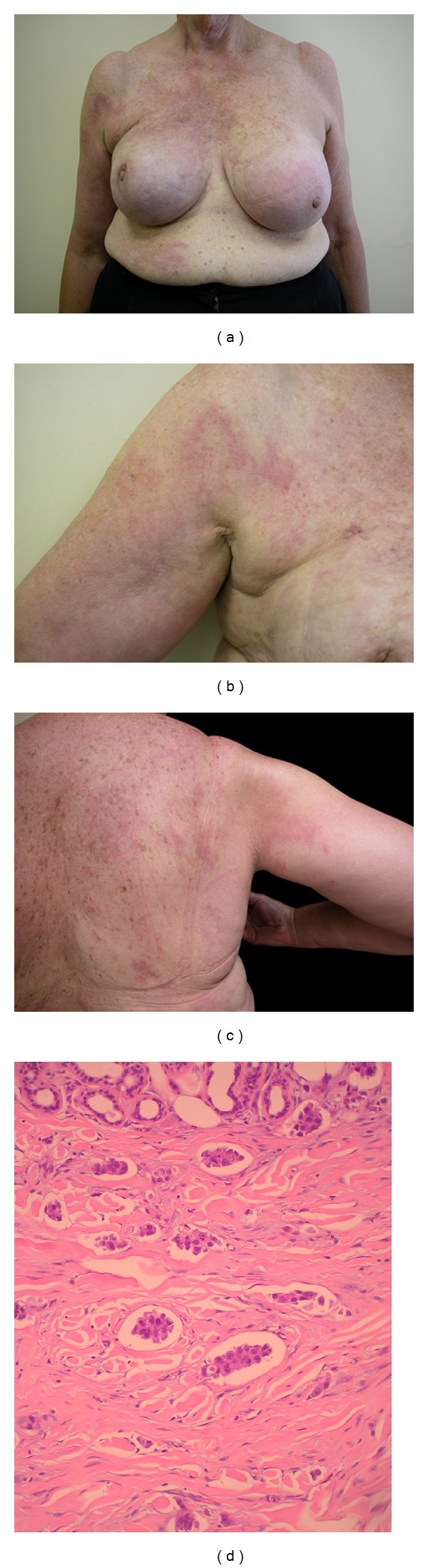
(a) The right breast is raised higher than the left breast and demonstrates nipple inversion. (b) Indurated erythema spreads across the right upper breast and upper arm. (c) Indurated erythema across the right upper back. (d) Extensive lymphatic invasion by infiltrative cords of epithelial cells.

A 62-year-old woman with a history of silicone breast augmentation presented with a nine-month history of erythema of the right arm and upper chest. A breast review had been undertaken, and an ultrasound was normal. She had previously been admitted twice to another hospital with a provisional diagnosis of cellulitis of her right arm, however, remained unresponsive to antibiotic therapy, prompting referral to dermatology by her local doctor. On examination, the right breast was raised higher than the left, the right nipple was inverted, and indurated erythema extended from the upper R breast to the upper arm and back (Figures 1(a), 1(b), and 1(c)). She had been examined previously on numerous occasions either wearing her bra or her nightdress in hospital. One specialist had ordered a breast ultrasound, which was normal. Carcinoma erysipelatoides was immediately suspected clinically. Skin biopsies of the indurated red areas on the chest and arm showed extensive infiltrative cords of epithelioid cells with ductal differentiation and lymphatic invasion, consistent with infiltrative breast carcinoma (Figure 1(d)). This patient was initially treated with Adriamycin/Cyclophosphamide chemotherapy followed by Docetaxel and Trastuzumab as she had Stage 4 disease at diagnosis including liver and boney metastasis. She developed cardiomyopathy secondary to Trastuzumab that was aggressively managed with some recovery of cardiac function using ACE inhibitors and digitalis that allowed reinstitution of Trastuzumab therapy. She then received several lines of chemotherapy sequentially for ongoing active disease including Capecitabine, Vinorelbine, Gemcitabine, and Metronomic Therapy with cyclophosphamide and Methotrexate. She died 23 months after her diagnosis. 

### 2.2. Case 2

**Figure 2:**
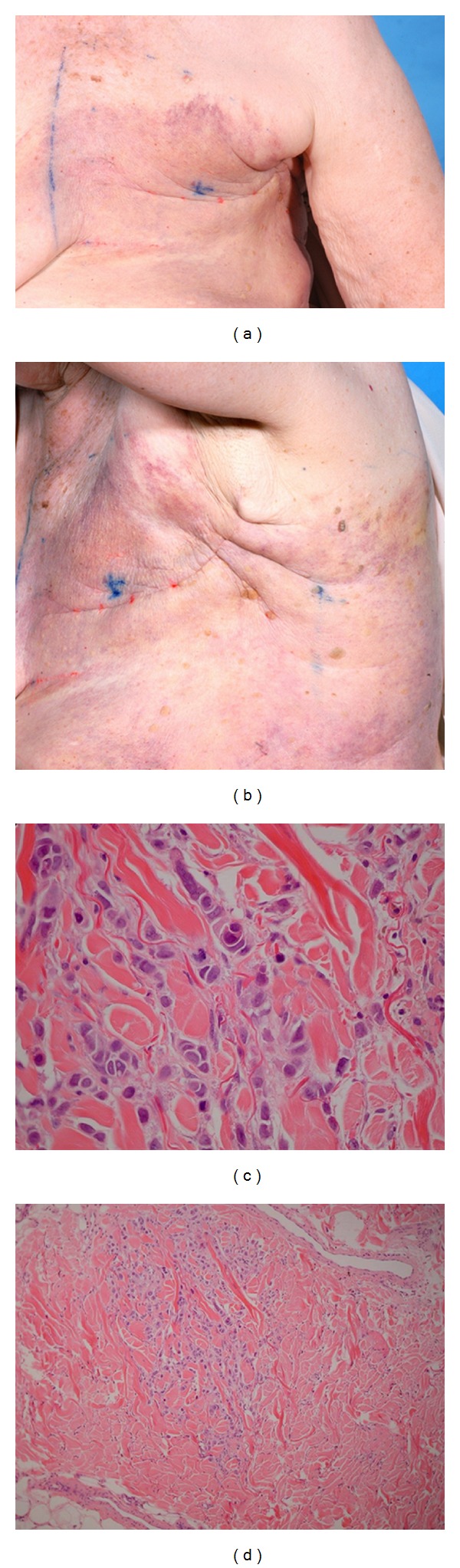
(a) Indurated left mastectomy scar with diffuse erythema and purpura. (b) The skin changes extend into the left axilla and back. (c) Groups of cells with pleomorphic nuclei and pale eosinophilic cytoplasm infiltrating dermal collagen. (d) Dermal infiltration by carcinoma.

A 75-year-old woman presented with a one-month history of erythema and induration of the left chest wall. Three months previously, she had undergone left mastectomy with adjuvant chemotherapy for locally advanced breast carcinoma and was due to begin radiotherapy. The patient was unresponsive to antibiotic treatment for a provisional diagnosis of cellulitis, prompting referral to dermatology. On examination, there was diffuse erythema and areas of purpura on the left chest wall extending to the left back, with induration over the mastectomy scar (Figures 2(a) and 2(b)). Three initial skin biopsies showed inflammation only; due to the dermatologists' concern, deeper sections were taken, and a small number of malignant cells were seen. Further skin biopsies revealed infiltrating adenocarcinoma in the dermis (Figures 2(c) and 2(d)). Initially, this patient was treated with single-agent Trastuzumab as the tumour was Her2+ with excellent clinical response and complete resolution of the rash. Unfortunately, she died following aspiration pneumonia following a CVA one year after her diagnosis.

### 2.3. Case 3

**Figure 3:**
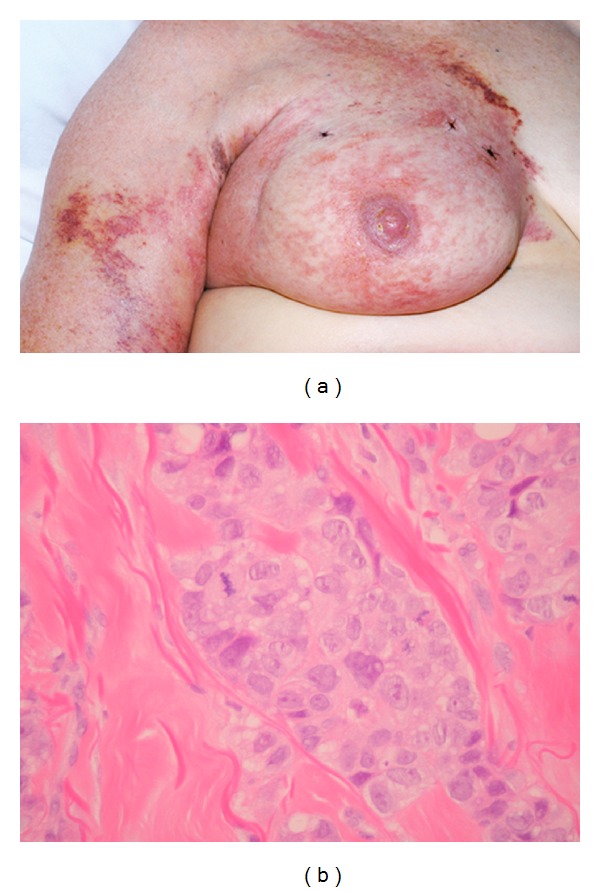
(a) The right breast displays patchy erythema with purpuric borders. (b) High-grade invasive ductal carcinoma infiltrating dermis, with mitotic figures.

A 72-year-old woman presented with a 1-week history of skin erythema over her right breast and right forearm, with worsening lymphoedema. She had a history of right breast invasive carcinoma five years previously, initially treated with lumpectomy, axillary dissection, and adjuvant chemoradiotherapy. Subsequent recurrence of an axillary mass was treated with palliative chemoradiotherapy, with minor response. On examination, the right breast, with lumpectomy scar, displayed patchy erythema with purpuric borders at the sternum, medial aspect of the upper right arm, and over the right scapula. Scattered within the erythema, there were palpable small nodules. Four skin biopsies showed widespread infiltration of the dermis by metastatic adenocarcinoma (Figures 3(a) and 3(b)). She subsequently received 4 lines of chemotherapy sequentially Docetaxel/capecitabine for 6 cycles, Gemcitabine/paclitaxel × 6 cycles, metronomic chemotherapy with cyclophosphamide/methotrexate, and single-agent carboplatin with no response and progressive skin involvement. She developed progressive pleural disease and died 18 months after her skin biopsy.

### 2.4. Case 4

**Figure 4:**
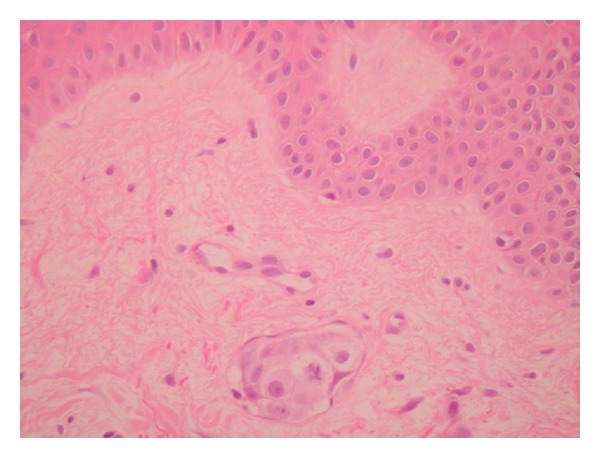
Focus of dermal lymphatic invasion with a mitotic figure.

A 53-year-old woman had a history of bilateral hormone-receptor-positive breast carcinoma, treated with double mastectomies, axillary clearances, adjuvant chemoradiotherapy, and hormonal therapy. Four years later, she developed multiple skeletal metastases, liver lesion, and mediastinal lymphadenopathy—at this point, therapy was switched from tamoxifen to anastrozole. She presented with a pathological fracture of the right humerus and several weeks of persisting erythema over the left breast and upper arm which was unchanged despite antibiotics, topical, and systemic corticosteroids. On examination, bilateral mastectomies and a port-a-cath were noted. There was patchy indurated erythema over the left breast and nonspecific blanching rash on the lateral aspect of the left upper arm. Biopsies from both regions showed infiltration of dermal lymphatics by metastatic ductal carcinoma, which was hormone receptor negative (Figure 4). Her performance status was poor as she had intercurrent pericardial disease with extensive bony disease, and she was treated with Gemcitabine/paclitaxel × 2 cycles followed by metronomic therapy and died 3 months following her diagnosis of superior vena cava obstruction with pericardial effusion and liver failure.

## 3. Discussion and Conclusion

The diagnosis of CE is made on clinical and/or pathological grounds. The clinical features, first established by Haagensen in 1971 [4] and incorporated by the American Joint Committee on Cancer (AJCC), are diffuse erythema, oedema involving 2/3 or more of the breast, peau d'orange, tenderness, induration, warmth, and enlargement—often without an underlying palpable breast mass. These findings are believed to be due to tumour invasion of dermal lymphatics, causing occlusion and “inflammation” [5].

Cutaneous metastases from primary internal organ malignancies are rare (3%–5% of patients with internal malignancies) [6], but when they do occur, the breast is the commonest primary site, in 24% of cases [7].

 CE is subdivided into a primary form, whereby carcinoma and inflammatory changes occur concurrently in a previously normal breast, and secondary, where inflammatory changes occur later in a breast with previous carcinoma—although both entities have similar survival rates [5]. Mention must also be made of its distinction from locally advanced breast carcinoma (LABC), which has no lymphatic vessel involvement (noninflammatory) and a slightly improved prognosis [8].

Once diagnosed, CE rapidly spreads, both locally and systemically. Trials have shown that between 46% and 100% of women have regional lymph node involvement at diagnosis [5], and approximately 36% have established distant metastases [9] commonly to bone, liver, and lung. Median disease-free survival is poor, at less than 2.5 years with multimodality treatment [9]. Poorer prognostic factors are axillary nodal involvement [5], younger age at diagnosis [8], African-American ethnicity [2], and negative hormone receptor status [5, 8]. Of note, the histology of cases 3 and 4 showed a “basal subtype” or “triple negative” (TN) breast cancer which is oestrogen receptor (ER), progesterone receptor (PR), and HER2 negative. TN breast cancers account for 15% of all newly diagnosed breast cancers and are associated with poorer survival and higher likelihood of metastases as they are not responsive to currently available targeted (hormonal) therapies. As IBC is frequently associated with a basal-like phenotype, it adds to the particularly adverse prognosis of this disease entity.

The rapid clinical onset and aggressive nature of CE requires that prompt diagnosis be made and therapy commenced as early as possible to improve survival. However, it often presents as a diagnostic challenge as it may be easily mistaken for other clinical entities. Commonly, these include: mastitis, cellulitis, erysipelas [7], thrombophlebitis, venous congestion [10], allergic reactions, postsurgical lymphoedema [11] or haematoma, postradiotherapy dermatitis, herpes zoster infection, primary squamous, or basal cell carcinoma [12]. In rarer cases, calciphylaxis [13] has been reported to mimic IBC. 

Diagnosis is confirmed by histologic sampling; however, this is not always reliable due to the variability of dermal lymphatic invasion, reported to be present in specimens obtained in 75% of patients only [1]. Thus, repeated sampling at multiple dermal levels should be done if IBC is strongly suspected clinically. Mammography has also shown to be helpful in providing important clues to the diagnosis, by showing features such as skin and stromal thickening and increased breast density, occasionally with a demonstrated mass or microcalcifications [3]. 

Here, we present a case series of 5 of our patients, some of whom had delay in diagnosis due to lack of recognition that a cellulites-like appearance may actually be metastatic breast cancer.

Treatment of CE previously consisted of surgery alone; however, this produced poor 5-year survival rates reported at less than 10% [5]. Mastectomy is currently preferred over breast conserving surgery but only yields an overall survival of 12 to 32 months when used alone. Adjuvant radiotherapy has been shown to improve locoregional tumour control but does not affect survival rates [5, 12]. Multimodality therapy is now the preferred option, reflecting the belief that systemic micro- or macrometastases are already present at diagnosis [5, 14] and is the cause of ensuing death. Chemotherapy, neoadjuvant and/or postoperative, can improve overall survival rates from less than 10% to 30%–55% when added with local modalities such as surgery and/or radiotherapy [1]. The role of hormonal therapy in IBC is variable as the majority of IBC cases tend to be oestrogen and progesterone receptor negative [9]. Overall, the inherently low incidence of IBC and lack of prospective trials make it difficult to establish a universal protocol for best treatment [1]. 

Evidence suggests that response to induction chemotherapy is the most important prognostic factor in disease-free and overall survival [15]. In addition, if initial presentation is with a palpable breast mass rather than diffuse involvement, this clinical entity tends to have a slightly improved prognosis—this is because a breast mass is more likely to be discovered and diagnosed earlier. Thus, reiterating our message, diagnosis of this deadly disease must be prompt in order to instigate early chemotherapy which may improve patient outcomes.

The previous cases demonstrate that CE can present insidiously and elude diagnosis both clinically and pathologically. Postsurgery for breast cancer, however, may occur not long afterwards and be confused with postoperative cellulitis/inflammation. It should always be a primary differential diagnosis for unilateral chest wall or arm erythema and induration in a history of malignancy. The lesson for clinicians is to ensure that the patient's upper body is always fully undressed and properly examined. The threshold for a tissue diagnosis should be low if there is no resolution with initial treatment. Multiple deeper sections of the initial biopsy or repeat skin biopsies should be performed if the pathology does not correlate with clinical findings.
